# Nell-1, a key Functional Mediator of Runx2, Partially Rescues Calvarial Defects in *Runx2*^+/−^ Mice

**DOI:** 10.1002/jbmr.267

**Published:** 2010-10-11

**Authors:** Xinli Zhang, Kang Ting, Catherine M Bessette, Cymbeline T Culiat, Sang Jin Sung, Haofu Lee, Feng Chen, Jia Shen, James J Wang, Shun'ichi Kuroda, Chia Soo

**Affiliations:** 1 Dental and Craniofacial Research Institute, University of California Los AngelesLos Angeles, CA, USA; 2 Section of Orthodontics, School of Dentistry, University of California Los AngelesLos Angeles, CA, USA; 3 Orthopaedic Surgery, School of Medicine, University of California Los AngelesLos Angeles, CA, USA; 4 Department of Bioengineering, University of California Los AngelesLos Angeles, CA, USA; 5 Oak Ridge National LaboratoryOak Ridge, TN, USA; 6 Department of Orthodontics, University of Ulsan College of Medicine, Asan Medical CenterSeoul, South Korea; 7 Department of Industrial Biosciences, Graduate School of Bioagricultural Sciences, Nagoya UniversityNagoya, Japan

**Keywords:** NELL-1, RUNX2, TRANSGENIC ANIMAL, CRANIOFACIAL DEVELOPMENT, CLEIDOCRANIAL DYSPLASIA

## Abstract

Mesenchymal stem cell commitment to an osteoprogenitor lineage requires the activity of Runx2, a molecule implicated in the etiopathology of multiple congenital craniofacial anomalies. Through promoter analyses, we have recently identified a new direct transcriptional target of Runx2, Nell-1, a craniosynostosis (CS)–associated molecule with potent osteogenic properties. This study investigated the mechanistic and functional relationship between Nell-1 and Runx2 in regulating osteoblast differentiation. The results showed that spatiotemporal distribution and expression levels of Nell-1 correlated closely with those of endogenous Runx2 during craniofacial development. Phenotypically, cross-mating *Nell-1* overexpression transgenic (*CMV-Nell-1*) mice with *Runx2* haploinsufficient (*Runx2*^*+/−*^) mice partially rescued the calvarial defects in the cleidocranial dysplasia (CCD)–like phenotype of *Runx2*^*+/−*^ mice, whereas Nell-1 protein induced mineralization and bone formation in *Runx2*^*+/−*^ but not *Runx2*^*−/−*^ calvarial explants. Runx2-mediated osteoblastic gene expression and/or mineralization was severely reduced by *Nell-1* siRNA oligos transfection into *Runx2*^*+/+*^ newborn mouse calvarial cells (NMCCs) or in *N*-ethyl-*N*-nitrosourea (ENU)–induced *Nell-1*^*−/−*^ NMCCs. Meanwhile, *Nell-1* overexpression partially rescued osteoblastic gene expression but not mineralization in *Runx2* null (*Runx2*^*−/−*^) NMCCs. Mechanistically, irrespective of Runx2 genotype, Nell-1 signaling activates ERK1/2 and JNK1 mitogen-activated protein kinase (MAPK) pathways in NMCCs and enhances Runx2 phosphorylation and activity when Runx2 is present. Collectively, these data demonstrate that Nell-1 is a critical downstream Runx2 functional mediator insofar as Runx2-regulated Nell-1 promotes osteoblastic differentiation through, in part, activation of MAPK and enhanced phosphorylation of Runx2, and Runx2 activity is significantly reduced when *Nell-1* is blocked or absent. © 2011 American Society for Bone and Mineral Research.

## Introduction

Commitment of undifferentiated mesenchymal stem cells to an osteoprogenitor lineage is first marked by expression of runt-related transcription factor 2 (Runx2; also known as Pebp2αA/Cbfa1/Aml3).(1,2) Runx2 is essential for osteoblast formation and function because it is expressed by all osteoblasts irrespective of embryonic origin or mode of ossification.(3) *Runx2* null mutant mice completely lack mineralized bone formation, whereas heterozygous *Runx2* loss-of-function mice manifest a phenotype similar to cleidocranial dysplasia (CCD) in humans, consisting of clavicular hypoplasia, delayed development and ossification of cranial bones causing open anterior and posterior fontanelles, smaller parietal and interparietal cranial bones, and multiple wormian bones (small bones in the sutures).(4,5) Since Runx2 is a transcription factor, it undoubtedly exerts its critical osteogenic effects in part through downstream functional mediators.(6) However, knockout models of many osteoblastic genes containing the consensus RUNX2 binding site osteoblast-specific binding elements 2 (OSE2), such as α*1 type I collagen*,(7) *bone sialoprotein*,(8) *osteopontin*,(9) and *osteocalcin*,(10) in mice have not yielded significant defects similar to Runx2 deficiency. Our data indicates that Nell-1 may be a key player in addition to Osx, another critical transcriptional factor for osteoblasts, in the Runx2 network regulating osteoblastic differentiation.(11–13)

NELL-1, a secreted protein strongly expressed in *n*eural tissues and containing *e*pidermal growth factor (EGF)–*l*ike domains (Nel)–*l*ike protein type *1*, was detected originally to be upregulated in pathologically fusing and fused sutures in *nonsyndromic* unilateral coronal synostosis (UCS) patients.(14) *Nell-1-*overexpressing mice (*CMV-Nell-1*) exhibit craniosynostosis (CS)–like phenotypes that ranged from simple to compound synostoses.(12) Through promoter analyses, we have established NELL-1 as a direct target of *RUNX2*, the master gene of osteochondrogenic differentiation.(6,15) The restoration of *Nell-1* mRNA expression after Runx2 transfection into *Runx2*^*−/−*^ cells indirectly confirms the existence of functional OSE2 binding sites in mouse *Nell-1* promoter and further supports *in silico* analysis findings of NELL-1 transcriptional regulation by RUNX2.(15) Furthermore, ENU-induced *Nell-1-*deficient mice display similar CCD-like calvarial phenotypes as *Runx2*^*+/−*^ mice in addition to rib cage and vertebral abnormalities.(16) The fact that RUNX2 directly promotes *NELL-1* transcripts and ENU *Nell-1-*deficient mice exhibit a similar CCD-like phenotype as *Runx2*^*+/−*^ mice suggests that NELL-1 may mediate a significant subset of downstream RUNX2 functions during osteoblastic differentiation in vivo.

The Nell-1 molecule itself is highly conserved across species. Rat and human Nell-1 proteins share a 93% predicted amino acid homology(14) and contain several conserved motifs.(17) More important, Nell-1 has revealed its osteoinductive potency by promoting bone regeneration in multiple animal models.(18–20) To better delineate the functional relationship between Runx2 and Nell-1 during skeletal development, we have used *Runx2-*deficient as well as *Nell-1-*deficient and -overexpressing mice models in this study. Because of the obvious calvarial abnormalities in both *Nell-1-*overexpressing and *Nell-1-*deficient mice, as well as the original identification of *NELL-1* upregulation in human UCS patients, we have focused our present osteoblast differentiation studies on intramembranous bone development, although Runx2 is also indispensable for normal chondrocyte hypertrophy and maturation.(21,22) Collectively, our data confirm for the first time that Nell-1 supports continued osteoblastic differentiation and function in osteoblastic lineage cells during calvarial development and that Nell-1 is a key functional mediator of Runx2 osteogenic activity.

## Materials and Methods

### Generation of *Runx2*-deficient plus *Nell-1* transgenic mice

*Runx2* heterozygous deficient mice (*Runx2*^*+/−*^)(4) were mated with *Nell-1-*overexpressing mice (*CMV-Nell-1*)(12) to generate *Runx2*^*+/−*^/*CMV-Nell-1* mice and *Runx2*^*−/−*^/*CMV-Nell-1* mice. Mouse genotypes were determined by PCR, and expression levels of *Nell-1* and *Runx2* were monitored using RT-PCR and were further verified by immunohistochemistry. Mouse embryos were collected from mating among wild-type mice with vaginal plugs defined as E0.5 days postcoitum (dpc). Table 1 lists the total number of animals used for skeletal staining, micro–computed tomography (µCT), and histology. Animals were housed and experiments were performed in accordance with guidelines of the Chancellor's Animal Research Committee of the Office for Protection of Research Subjects at the University of California Los Angeles.

**Table 1:** Summary of Animals Used for Detailed Analysis

		No. used for skeletal analyses			No. of animals “rescued” from Runx2 deficiency^a^
				
Genotype	Total no. of animals	No. used for staining	No. used for µCT	No. used for histology	
*Runx2*^*+/+*^*/*					
*CMV-Nell-1*	12	4	3	5	Not applicable
WT	15	2	3	10	Not applicable
*Runx2*^+/*−*^/					
*CMV-Nell-1*	27	15	7	5	16 of 22
WT	35	20	5	10	Not applicable
*Runx2*^*−/−*^/					
*CMV-Nell-1*	8	3	1	4	0 of 4
WT	9	5	1	3	Not applicable
Total	106	49	20	37	

a Determination of “rescue” status was based on skeletal staining and µCT analysis.

### Ex vivo calvarial organ culture

Calvarial vaults of newborn *Runx2*^*+/−*^ mice were harvested and placed in serum-free BGJb (Biggers, Gwatkin, Judah) medium with l-glutamine and supplemented with 100 unit/mL of penicillin, 100 µg/mL of streptomycin, 2.5 µg/mL of amphotericin B, 100 µg/mL of l-ascorbic acid, and 10 mM glycerophosphate. Recombinant Nell-1 (rNell-1) protein (Katayama Chemical, Ltd., Osaka, Japan) at 100 ng/mL was added beginning on day 1 [plus rNell-1 (*n* = 8) or minus rNell-1 (*n* = 5)]. On day 4, calcein was added to the culture medium at 2 µg/mL, and the explants were maintained for a total of 9 days before harvesting for gross and histologic analysis of tissue ossification. The calcein deposition on explants was observed with an Olympus SZX12 fluorescent microscope (Melville, NY, USA), and the relative intensity of green fluorescence representing the degree of mineralization on whole explanted calvaria as well as defined coronal and sagittal suture areas was quantified using Image Pro Plus (Bethesda, MD, USA). The methyl methacrylate–embedded sections were analyzed under an Olympus BX51 fluorescent microscope.

### Skeletal and histologic analysis

Newborn mice with the genotypes described in Table 1 were euthanized with an overdose of phenobarbital, skinned and eviscerated, and then fixed in 95% ethanol for 24 hours at room temperature. Standard skeletal staining was performed using alcian blue for negatively charged proteoglycans and alizarin red for calcium to provide gross distinction between cartilage and mineralized tissue, respectively. For histology, tissues were fixed in 4% paraformaldehyde, embedded in paraffin, and stained with hematoxylin and eosin (H&E). Skeletal and histologic images were acquired using a MicroFire digital camera with PictureFrame software (Optronics, Goleta, CA, USA) attached to Olympus SZX12 and BX51 microscopes.

### High-resolution µCT analysis

High-resolution µCT using 9- to 20-µm resolution technology from µCT40 (Scanco USA, Wayne, PA, USA) was performed as described previously.(18) µCT data were collected at 50 kVp and 160 µA and reconstructed using the cone-beam algorithm supplied with the µCT scanner by Scanco. A threshold of 130 for 3D reconstruction of newborn mouse heads was determined empirically by evaluating skeletal image of newborn wild-type mouse heads with serial thresholds to choose the one where no soft tissue was detected. In addition, CT-based morphometric analyses were performed on the size of the anterior fontanel, the closest distance of the sagittal suture, and the average thickness of parietal bone plates on the same plane in each group including *Runx2*^*+/+*^ (*n* = 3), *Runx2*^*+/−*^ (*n* = 5), and *Runx2*^*+/−*^*/CMV-Nell-1* (*n* = 7) newborn mice. Data are presented as the mean ± SD and analyzed with a two-tailed Student's *t* test, with *p* ≤ .05 considered significant.

### Immunohistochemistry

Whole-head samples from E14.5, E16.5, E18.5, and newborn mice were fixed in 4% paraformaldehyde and then processed for paraffin embedding. Paraffin-embedded sections were used for immunohistochemistry employing a previously described protocol.(12) Polyclonal rabbit IgG of anti-mouse Nell-1 antibodies were synthesized against an 11-amino-acid peptide, and the specificity of the affinity-purified antibody was further confirmed by Western blot using rNell-1. All other commercially available antibodies, including anti-Runx2, anti-osteocalcin (Ocn), and anti-osteopotin (Opn), were from Santa Cruz Biotechnology (Santa Cruz, CA, USA). ABC reagent was from Vector Lab (Burlingame, CA, USA), and Alex 594–streptavidin was from Invitrogen (Carlsbad, CA, USA). Negative controls for each antibody were included and performed accordingly with the absence of primary antibody.

### Isolation and culture of NMCCs

Isolation of *Runx2*^*−/−*^ or ENU-induced *Nell-1*-deficient(16) newborn mouse calvarial cells (NMCCs; *n* = 5) as well as their corresponding wild-type (*n* = 10) or heterozygous NMCCs (*n* = 10) was conducted within 2 hours of delivery of mice, as reported previously.(15) The NMCCs were cultured in α minimum essential medium (α-MEM) supplemented with 10% fetal bovine serum (Life Technologies, Inc., Grand Island, NY, USA), 100 units/mL of penicillin, and 100 µg/mL of streptomycin. Cell expansion was limited to three passages, and the genotype of isolated NMCCs was further confirmed by PCR or RT-PCR and immunocytochemistry. NMCCs at third passage were used in all experiments.

### Real-time RT-PCR

TaqMan primer probe sets for *Alp*, *Opn*, *Ocn*, *Nell-1*, *Runx2*, and *glyceraldehyde-3-phosphate dehydrogenase* (*Gapdh*) were purchased (Applied Biosystems, Inc., Foster City, CA, USA) and analyzed using an ABI Prism 7300 real-time reverse-transcriptase (RT) PCR system (Applied Biosystems) as described previously.(23) Relative gene expression profiles were calculated using the comparative quantification formula as 2^*−*ΔΔ*Ct*^ based on the evaluation of similar dynamic ranges for RT-PCR efficiency of both *Gapdh* and the target genes. All data are representative of three experimental sets of cells or three mice tissue specimens with duplicate PCR running and are presented as the fold difference. In addition, the Northern blot hybridization and reduced-cycle RT-PCR also were used for the detection of endogenous levels of *Runx2*, *Nell-1*, *Ocn*, *Osx*, *Dlx5*, *and Msx2* in whole-head tissues of mice with different genotypes, as described previously.(13) PCR primer sequences are available on request.

### Western blot detection of MAPK pathways activation

NMCCs were synchronized for 18 hours in 0.1% fetal bovine surum (FBS) growth medium before being stimulated with 100 ng/mL of rNell-1 for the indicated times. The cells were lysed in radioimmunoprecipitation assay (RIPA) buffer supplemented with inhibitors of proteinase and phosphatase (Invitrogen) and underwent Western blot analysis with antibodies against total and phosphorylated ERK, JNK and p38 (Santa Cruz Biotechnology) using previously reported protocols.(13)

### Immunoprecipitation for Runx2 phosphorylation study

NMCCs were synchronized as described earlier and preincubated for 1 hour with three MAPK inhibitors at 25 µM SP600125 for JNK, 50 µM PD98059 for ERK, and 10 µM SB203580 for p38 or equal volume of DMSO vehicle in 0.1% FBS growth medium before being stimulated with 100 ng/mL of rNell-1 for 1 hour. Immunoprecipitation was done by adding 2 µg of anti-Runx2 (Abcam, Cambridge, MA, USA) to 500 µL of cell lysate and incubating for 1 hour at 4°C, followed by mixing with 50 µL of protein G sepharose. The immunoprecipitation products were probed with anti-Runx2 and anti-pSer (Santa Cruz Biotechnology) for detecting Runx2 phosphorylation status.

### Reporter assay for transactivation analysis

The reporter assay was done by cotransfection of plasmids of 6OSE2 plus *Renilla* control with either Runx2 expression plasmid pcDNA-Runx2 or empty-vector pcDNA3.1 into *Runx2*^*+/−*^ and *Runx2*^*−/−*^ NMCCs using Lipofectamin 2000 (Invitrogen). Then 100 ng/mL or higher at 800 and 1600 ng/mL of rNell-1 and equal volumes of PBS as control were added to medium 24 hours after transfection, and the medium was incubated for another 24 hours before collecting cell lysate for luciferase assay using a dual luciferase detection kit (Promega, San Luis Obispo, CA, USA) as described previously.(15)

### Adenoviral transduction with NMCCs

NMCCs at 80% confluence were transduced with Ad*Nell-1*, Ad*LacZ*, or Ad*Runx2* at a multiplicity of infection (MOI) of 50 plaque-forming units (pfu) per cell in α-MEM, as described previously.(13) Alkaline phosphatase activity and the expression of *Nell-1*, *Alp*, *Opn*, and *Ocn* and/or mineralization were analyzed at the indicated time points.

### Transfection of *Nell-1* siRNA into NMCCs

Mouse *Nell-1* siRNA oligos were designed and synthesized by Qiagen with the HiPerformance Design Alogrithm based on the sequences of mouse *Nell-1* (Gene Accession No. AK046127). The specific target sequence was as follows: CAGGTGTGGATTCTGAGAGAA. RNAifect reagent and unrelated negative control siRNA (Qiagen) were used for the transfection of siRNA into NMCCs according to the manufacturer's instructions. Briefly, NMCCs were seeded at 5 × 10^4^/cm^2^ into 24-well plates and allowed to reach 80% confluence the following day for siRNA transfection. Then 50 ng of siRNA oligos per well was mixed with the Perfect reagent at a ratio of 1:3 and incubated for 10 minutes at room temperature. The blocking efficacy for the expression of mouse *Nell-1* mRNA and protein was measured at 48 and 72 hours after transfection with RT-PCR and Western blot analysis with Nell-1-specific antibody.(13) The transfection efficiency and blocking specificity of *Nell-1* siRNA oligos into these cells was monitored by introducing unrelated siRNA oligos labeled with fluorescein using the same strategy. For some groups, the transfection of mouse *Nell-1* siRNA oligos was followed by the transduction of Ad*Runx2*(24) into the same cells 24 hours later. Seventy-two hours after seeding, cells were switched to differentiation medium containing 50 µg/mL of ascorbic acid and 10 mM glycerol phosphate. The Western blot for Nell-1, real-time PCR for *Nell-1*, *Runx2*, *Alp*, *Opn*, and *Ocn* mRNA, and von Kossa staining were carried out accordingly.

### ALP and mineralization assays

Alkaline phosphatase (ALP) staining assay was carried out with the Leukocytes ALP Staining Kit (86R-1KT, Sigma, St Louis, MO, USA) as described previously.(13) Quantitative ALP activity was assayed with cell lysate and ALP Buffer Solution (Sigma) and phosphatase substrate capsule (Sigma), as reported previously.(20) All measurements were read in triplicate, and the ALP activity was normalized to corresponding protein quantifications before making relative fold determination against nontransduced control. The data are presented as the mean ± SD with a *t-*test significance of *p* ≤ .05. The von Kossa and alizarin red staining (ARS)/quantification assays were performed as described previously.(12,20)

### Cell proliferation and apoptosis analysis

To determine nonspecific toxicity of MAPK inhibitors, NMCCs were seeded at 2 × 10^3^ cells/well in 96-well plates in the presence of MAPK inhibitors or DMSO vehicle for proliferation study by the MTT [3-(4,5-dimethyl-2-thiazolyl)-2,5-diphenyl-2H-tetrazolium bromide] method (Promega). The optical density (OD) values at 490 nm were plotted for relative comparison among different treatment groups. For apoptosis analysis, NMCCs were plated in 6-well culture dishes and treated with MAPK inhibitors at the same concentration used for the *Runx2* phosphorylation study for 6 days in osteoblastic differentiation medium. The Annexin V and propidium iodide (PI) Kit (Pharmingen, San Diego, CA, USA) was used for staining cells, followed by flow cytometric analysis. The percentage of early and late apoptotic cells was calculated and presented.

## Results

### Spatiotemporal expression of Nell-1 and Runx2 during mouse craniofacial bone development

**Fig. 1:**
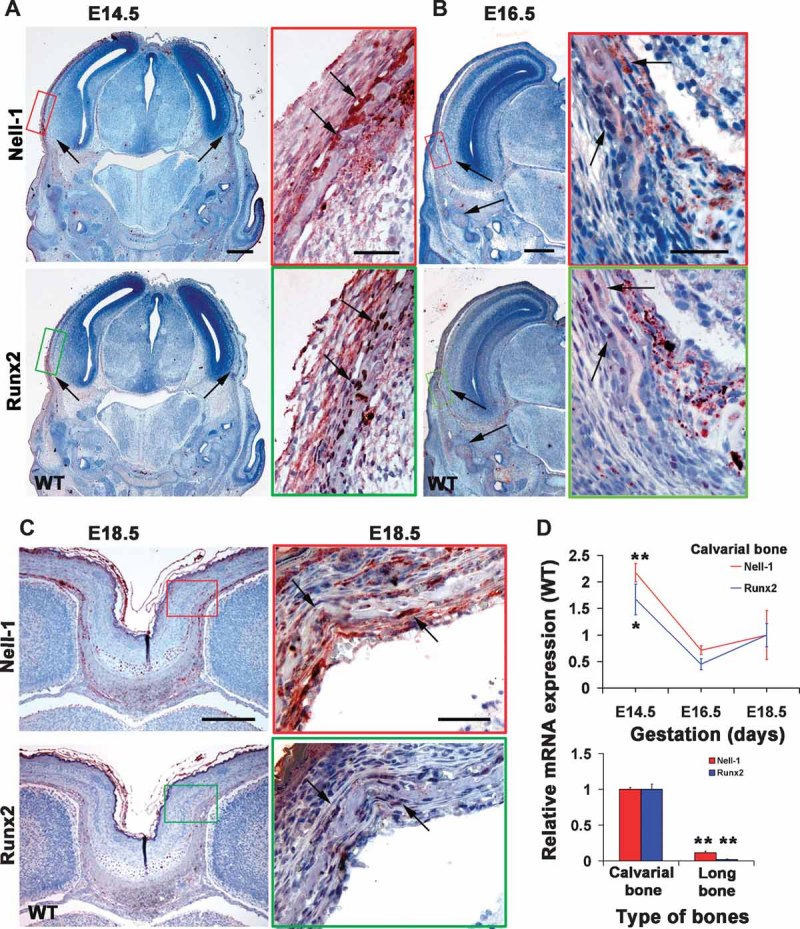
Spatiotemporal expression of Runx2 and Nell-1 during mouse craniofacial development. (*A*) E14.5, (*B*) E16.5, and (*C*) E18.5 mice craniofacial tissues were treated with Nell-1 (*upper panel*) or Runx2 (*lower panel*) antibody for immunohistochemistry. Low-magnification (*left*) and high-magnification (*right, color framed*) images demonstrate cellular localization and tissue distribution of Nell-1 and Runx2 in embryos at different stages. Arrows indicate areas of positive staining. Nuclear localization of Runx2 and prominent cytoplasmic staining of Nell-1 were clearly displayed in osteoblast cells along the temporal bone plate in E14.5 and E16.5 embryos as well as in osteoblast cells along parietal bone plates in E18.5 embryos. Scale bar: 500 µm for low-magnification and 50 µm for high-magnification images in panels *A* and *B*; 250 µm for low-magnification and 50 µm for high-magnification images in panel *C*. (*D*) Graphic depiction of calvarial bone *Nell-1* (*red line*) and *Runx2* (*blue line*) mRNA expression during embryonic development (*top*). Bar graph depiction of significantly higher *Nell-1* (in *red*) and *Runx2* (in *blue*) mRNA expression in calvaria versus long bone in newborn animals (*bottom*) by real-time RT-PCR. *p < .05; **p < .01.

To determine the spatiotemporal expression pattern of Runx2 and Nell-1 during craniofacial bone formation, immunohistochemistry was performed on whole-head sections of E14.5, E16.5, and E18.5 mouse embryos. Consistent with its critical role in osteoblastogenesis, Runx2 expression was present in every axial, appendicular, and cranial skeletal anlage at E12.5,(3) although mineralization of craniofacial bones occurs later, at E14.5. In E14.5 mice, significantly high levels of Nell-1 expression closely approximated and overlapped with Runx2 expression in temporal and parietal bone plates (Fig. 1*A*). Osteoblast cells along the temporal bone plates with typical nucleus staining for Runx2 (Fig. 1*A*, *lower panel*) also stained strongly for Nell-1 in the cytoplasm and nucleus (Fig. 1*A*, *upper panel*). In E16.5 mice, the spatial distribution of Nell-1 expression expanded to cover bone-forming areas of the cranial vault, base, and mandible (Fig. 1*B*). High-intensity Nell-1-positive cells localized primarily to ossifying membranous bone-forming regions in the cranial base as well as nonossifying regions such as the dura mater (Fig. 1*B*, *upper panel*). Interestingly, in E18.5 mice, Runx2 expression was relatively low in calvarial bone and dura mater when compared with Nell-1 expression (Fig. 1*C*). To complement the immunohistochemistry data, relative *Runx2* and *Nell-1* mRNA levels in isolated E14.5, E16.5, and E18.5 calvarial bones were determined by real-time RT-PCR. *Nell-1* expression closely paralleled *Runx2* mRNA expression with a significant decline in levels from E14.5 to E16.5 (Fig. 1*D*, *upper panel*). Overall, the craniofacial localization and expression pattern of Nell-1 followed that of Runx2. The close temporal and spatial overlap in Runx2 and Nell-1 expression at multiple embryonic stages suggests a possible regulatory relationship between Runx2 and Nell-1 during mouse craniofacial development.

To determine if the observed similarity in *Runx2* and *Nell-1* expression patterns in calvarial bones also were present in long bones, neonatal calvaria and long bone (femur and tibia) RNA were analyzed by real-time RT-PCR. Results demonstrated that *Runx2* levels in newborn calvarial bone were higher than in newborn long bone. Relatively high *Runx2* levels in calvaria were associated with high calvarial *Nell-1* levels, whereas lower *Runx2* levels in long bone were associated with lower *Nell-1* levels (Fig. 1*D*, *lower panel*). These data demonstrated closely paralleled expression patterns for *Nell-1* and *Runx2* in both calvarial and long bone tissues, as well as significantly higher *Nell-1* and *Runx2* RNA levels in calvaria versus long bones during development.

### Diminished expression of *Nell-1* in craniofacial tissues with *Runx2* deficiency

**Fig. 2:**
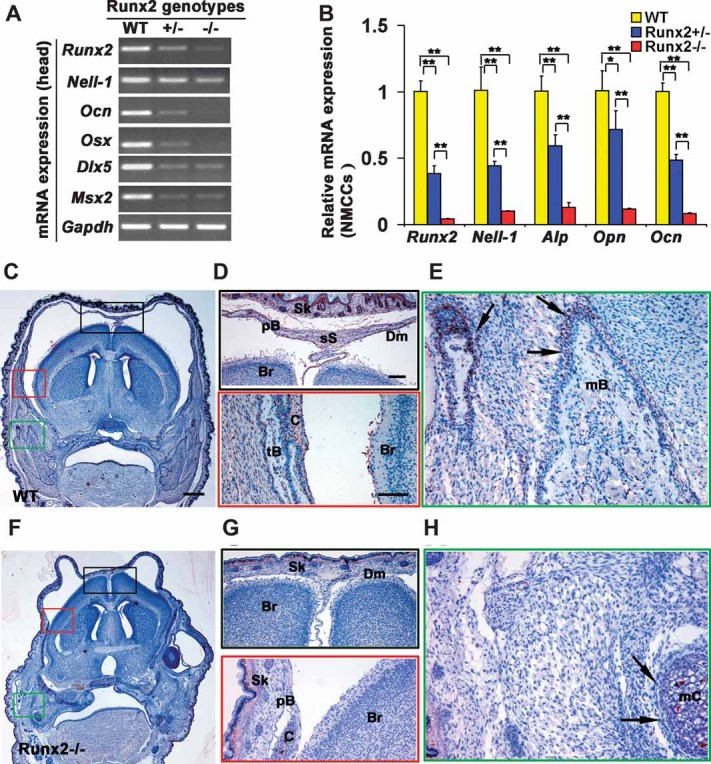
*Nell-1* mRNA and protein levels in craniofacial tissues of newborn Runx2-deficient mice. (*A*) Reduced cycle RT-PCR on *Runx2*, *Nell-1*, *Ocn*, *Osx*, *Dlx5*, *Msx2*, and *Gapdh* mRNA levels from whole-head tissues of newborn wild-type (WT), *Runx2* haploinsufficient (*Runx2^+/−^*), or *Runx2* null (*Runx2^−/−^*) mice. (*B*) Quantitative real-time RT-PCR analysis on *Runx2*, *Nell-1*, *Alp*, *Opn*, and *Ocn* mRNA levels from NMCCs (newborn mouse calvarial cells isolated from 10 WT, *10 Runx2^+/−^*, and 5 *Runx2^−/−^* mice) with WT (*yellow*), *Runx2^+/−^* (*blue*), or *Runx2^−/−^* (*orange*) genotypes. *p < .05; **p < .01. (*C*) Nell-1 immunohistochemistry on coronal sections from WT newborn mouse head at low magnification (scale bar: 500 µm) and corresponding high-magnification areas at (*D*) the highlighted sagittal suture (*upper, black border*; scale bar: 100 µm), temporal bone (*lower, red border*; scale bar: 100 µm) and (*E*) the mandible (*green border*; scale bar: 50 µm). Significant positive Nell-1 staining is evident across the section, and readily identifiable positive cells are seen at the calvarial sagittal suture (sS), parietal bone (pB), dura mater (Dm), and scalp (Sk). Prominent positive staining also was present at the periosteum (*arrows*) of mandibular bone (mB). (*F*) Nell-1 immunohistochemistry on coronal sections from a newborn *Runx2^−/−^* mouse head. Nell-1 protein was undetectable in the *Runx2^−/−^* mouse head (scale bar: 500 µm) in contrast to its WT littermates. Corresponding (*G*) sagittal suture (*upper, black border*; scale bar: 100 µm), temporal bone (*lower, red border*; scale bar: 100 µm) and (*H*) mandible (*green border*; scale bar: 50 µm) areas show distinct absence of bone development and no Nell-1 staining. Nell-1 staining in *Runx2^−/−^* animals was present primarily in skin (*G*) and a few chondrocytes (*H*) (*arrows*) in Meckel's cartilage (mC).

The preferential expression of *Nell-1* and *Runx2* in calvaria versus long bones and their spatiotemporal distribution pattern in craniofacial bones led us to further investigate the relationship between Runx2 and Nell-1 in the context of *Runx2* disruption. Whole-head tissues from wild-type (*Runx2*^*+/+*^), *Runx2*^*+/−*^, and *Runx2*^*−/−*^ newborn mice were examined for expression of *Nell-1* and *Ocn* by Northern blot (Supplemental Fig. S1*A*). The highest expression of an approximately 3.5-kb single transcript of *Nell-1* and an approximately 0.7-kb *Ocn* was readily detected in *Runx2*^*+/+*^ tissue, and only weak signals were observed in *Runx2*^*+/−*^, whereas both *Nell-1* and *Ocn* were undetectable in all *Runx2*^*−/−*^ tissues. In addition, the expression levels of *Nell-1* and *Ocn*, as well as *Runx2* and other genes critical in craniofacial development, including *Osx*, *Dlx5*, and *Msx2*, were verified and screened with reduced-cycle RT-PCR using the same RNAs from whole-head tissues used for Northern blot analysis (Fig. 2*A*). Results revealed that the levels of *Runx2* expression in *Runx2*^*+/+*^, *Runx2*^*+/−*^, and *Runx2*^*−/−*^ samples correlated very well with the expression levels of *Nell-1*, *Ocn*, and *Osx*, whereas the levels of *Dlx5* and *Msx2* were similar in both *Runx2*^*+/−*^ and *Runx2*^*−/−*^ samples. These results indicate that *Nell-1*, *Ocn*, and *Osx* gene transactivation is highly dependent on underlying Runx2 levels.

To specifically correlate *Runx2* and *Nell-1* expression in bone-forming tissues, the gene expression levels of *Runx2*, *Nell-1*, *Ocn*, *Opn*, and *Alp* were further analyzed with newborn mouse calvarial cells (NMCCs) (Fig. 2*B*) by real-time RT-PCR, and the protein level of Nell-1 in situ was detected in *Runx2*^*+/+*^ and *Runx2*^*−/−*^ craniofacial tissue sections by immunohistochemistry (Fig. 2*C–H*). *Nell-1* expression levels in NMCC correlated well with *Runx2* genotypes and mRNA levels of *Runx2*, *Alp*, *Opn*, and *Ocn*; the highest *Nell-1* levels were present in *Runx2*^*+/+*^ cells, intermediate levels were seen in *Runx2*^*+/−*^ cells, and there were barely detectable levels in *Runx2*^*−/−*^ cells. Nell-1 immunohistochemistry on *Runx2*^*+/+*^ and *Runx2*^*−/−*^ newborn craniofacial tissues revealed greatly diminished Nell-1 staining intensity in *Runx2*^*−/−*^ mice calvarial tissue, dura mater, and mandibular bones compared with wild-type mice (Fig. 2*C–H*). Collectively, the close correlation between Nell-1 and Runx2 expression indicates that Runx2 is an important in vivo regulator of *Nell-1* expression and is consistent with our data demonstrating potentially functional Runx2 binding sites on the Nell-1 promoter.(15)

### Nell-1 partially rescues CCD-like calvarial defects in *Runx2*^+/*−*^ mice

**Fig. 3:**
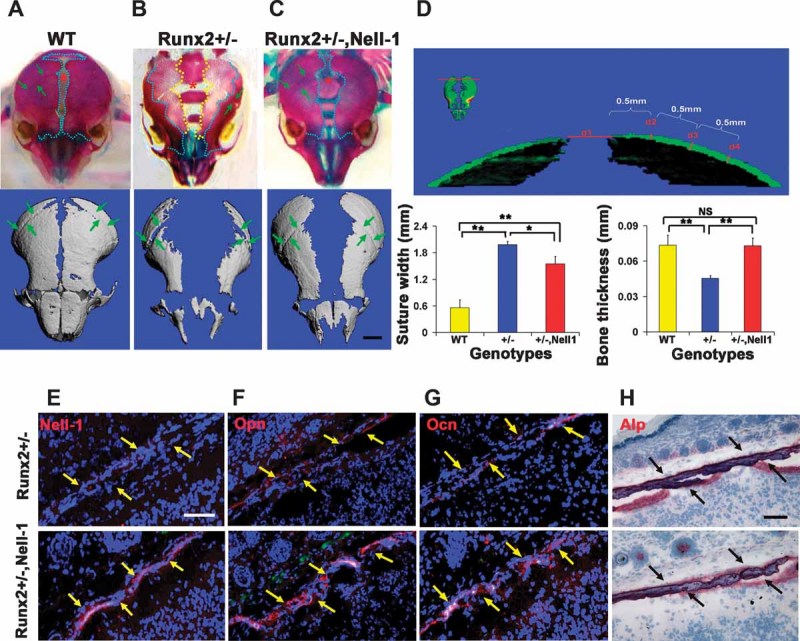
Skeletal and histochemical analysis of rescued CCD *Runx2^+/−^* newborn mice. (*A*) The skeletal staining (*top*) and µCT (*bottom*) of a wild-type mouse demonstrating typical mineralization borders (*dotted light blue line*) and the location of the anterior (*red asterisk*) calvarial fontanel. The coronal sutures (*green arrows*) also are highlighted. (*B*) *Runx2^+/−^* animal demonstrating widely patent midline sutures and fontanels. Defective mineralization and bone formation are present in the poorly stained tissue (*between yellow and light-blue dotted lines*) lateral to the midline calvarial defect. A lucency also can be seen in the area of the coronal suture (*green arrows, top and bottom pictures*). (*C*) *Runx2^+/−^*/*CMV-Nell-1* animal demonstrating significantly increased calvarial bone formation relative to the *Runx2^+/−^* animal on skeletal staining and µCT. Note the restoration of bony overlap at the coronal sutures (*green arrows*) and decreased anterior fontanel size (*red asterisk*). Scale bar: 1 mm for µCT images. (*D*) The red line is drawn through the location of parietal bones (*top, upper left image*) for cross-sectional analysis. Cross-sectional view with labels *d1* to *d4* (*top, center image*). The label *d1* represents sagittal suture width, whereas *d2* through *d4* are measurement points of parietal bone thickness starting from *d2* at 0.5-mm intervals. Quantitative analysis of the average width and thickness are depicted graphically (*bottom charts*, *p < .01; **p < .05; NS = no significance). (*E–G*) Immunohistochemistry of Nell-1, Opn, and Ocn as well as (*H*) Alp enzymatic histochemistry on calvarial bones. *Runx2^+/−^*/*CMV-Nell-1* tissues demonstrated markedly higher staining intensity (*red color*) for Nell-1, Opn, and Ocn (*yellow arrows*) relative to *Runx2^+/−^* tissues, whereas Alp activity did not show significant differences. Scale bar: 50 µm.

Global *Nell-1-*overexpressing mice using a CMV promoter (*CMV-Nell-1*) exhibit phenotypes related to calvarial overgrowth and premature suture fusion without obvious extracranial abnormalities (despite verification of global *Nell-1* transgene expression).(12) To determine whether *Nell-1* can functionally compensate for some aspects of *Runx2* deficiency (eg, CCD phenotype), *Runx2*^*+/−*^ mice were mated to *CMV-Nell-1* mice to generate *Runx2*^*+/−*^/*CMV-Nell-1* mice. A total of 106 newborn mice recovered from 14 litters were included in this study (Table 1). As expected, newborn wild-type mice showed dramatically different patterns of cranial bone skeletal staining compared with *Runx2*^*+/−*^ mice. Consistent with previous reports, the majority of *Runx2*^*+/−*^ mice exhibited developmental anomalies resembling the CCD phenotype described by Otto and colleagues (eg, hypoplastic clavicles, wide sutures, and delayed membranous bone ossification resulting in open anterior and posterior fontanelles as well as wide cranial sutures)(4) (Fig. 3*A, B*).

Remarkably, 16 of the 22 *Runx2*^*+/−*^/*CMV-Nell-1* mice demonstrated “rescue” of calvarial bone defects associated with the CCD phenotype on skeletal or µCT analyses (Fig. 3*C, D*), whereas Nell-1 did not rescue bone formation in the *Runx2* null mice (data not shown). Quantitative µCT data confirmed significant reduction of sagittal suture widths in *Runx2*^*+/−*^/*CMV-Nell-1* mice compared with *Runx2*^*+/−*^ mice (*p* < .05; Fig. 3*D*). Furthermore, parietal bone thickness in *Runx2*^*+/−*^/*CMV-Nell-1* mice was significantly increased relative to *Runx2*^*+/−*^ mice (*p* < .01) and comparable with wild-type (*Runx2*^*+/+*^) bone thickness (Fig. 3*D*). In addition, the anterior fontanelle openings in *Runx2*^*+/−*^/*CMV-Nell-1* mice were markedly smaller than in *Runx2*^*+/−*^ mice by visualization (Fig. 3*C*). Preliminary analysis of calvaria from P_21_ mice by µCT also revealed narrower sagittal sutures and increased intensity of calvarial bones in *Runx2*^*+/−*^/*CMV-Nell-1* mice compared with *Runx2*^*+/−*^ mice (Supplemental Fig. S1*B*). There was no rescue of clavicular hypoplasia because no significant difference was observed in clavicle length or thickness between *Runx2*^*+/−*^ and *Runx2*^*+/−*^/*CMV-Nell-1* mice (data not shown).

Immunohistochemistry clearly demonstrated increased Nell-1, Opn, and Ocn in calvarial bone plates of the rescued *Runx2*^*+/−*^/*CMV-Nell-1* mice (Fig. 3*E–G*) but not increased Alp (Fig. 3*H*). These data show that Nell-1 can functionally compensate for some aspects of *Runx2* deficiency and suggest that Nell-1 is a key downstream mediator of Runx2's effects on osteoblastic differentiation and bone formation.

### Nell-1 enhanced mineralization in ex vivo calvarial explants from *Runx2*^+/*−*^ mice

**Fig. 4:**
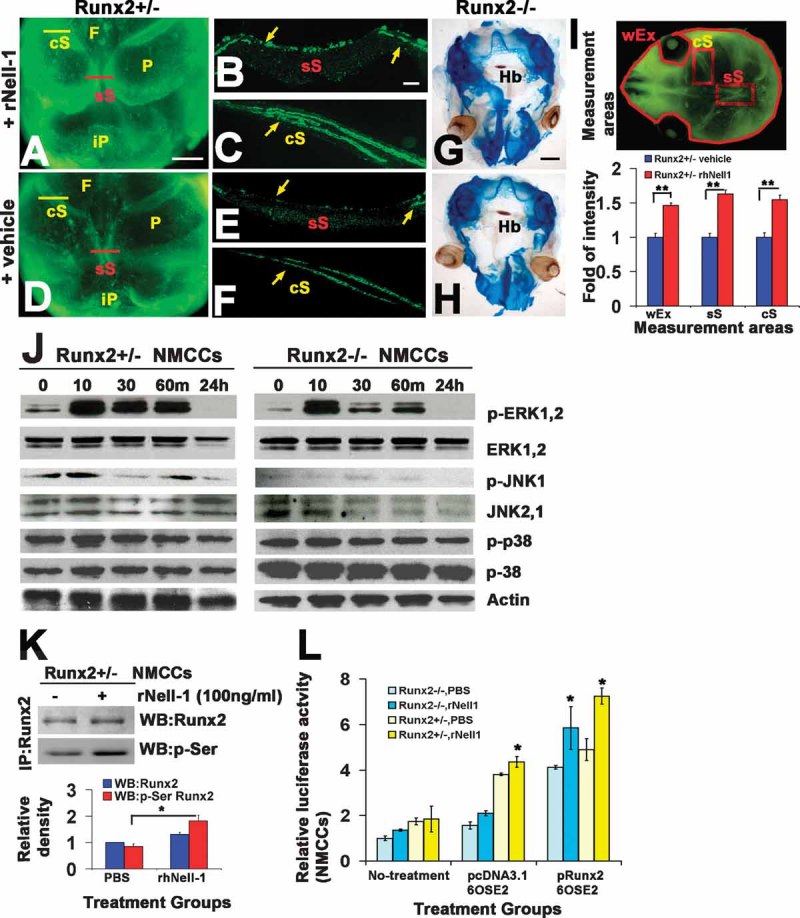
Nell-1 protein promotes *Runx2^+/−^* calvarial explant mineralization and activates MAPK pathways with NMCCs. (*A*) *Runx2^+/−^* calvarial explants with rNell-1 stimulation displayed intense calcein fluorescent labeling at the peripheral edges of parietal bones (P), coronal sutures (cS), and sagittal sutures (sS), indicating active mineralization. (*B*) Coronal section displaying fluorescent incorporation in a sagittal suture (sS) corresponding to the red line in panel *A*. rNell-1 induces significant mineralization in sagittal suture mesenchyme and osteogenic fronts of parietal bone (*yellow arrows*). (*C*) Coronal section displaying fluorescent incorporation in a coronal suture corresponding to the yellow line in panel *A*. Nell-1 induces marked new bone formation with resulting fusion of the overlapping frontal and parietal bone plates at the coronal suture (*yellow arrow*). (*D*) *Runx2^+/−^* calvarial explants without rNell-1 stimulation revealed reduced mineralization, as represented by green fluorescent staining throughout the entire calvarial vault. (*E*) Coronal section displaying fluorescent incorporation in a sagittal suture corresponding to the red line in panel *D*. Reduced sutural and osteogenic front mineralization were observed. (*F*) Coronal section displaying fluorescent incorporation in a coronal suture corresponding to the yellow line in panel *D*. A well-separated gap between the overlapping frontal and parietal bones demonstrating coronal suture patency (*yellow arrow*). (*G*) Skeletal staining of *Runx2^−/−^* calvarial explants (with brain attached) with rNell-1 or (*H*) without rNell-1 did not show any alizarin red staining except for hyoid bone (Hb). Scale bars: 500 µm for *A* and *D*; 50 µm for *B*, *C*, *E*, and *F*; 1 mm for *G* and *H*. (*I*) Quantitative measurement of mineralization intensity using ImageProPlus on whole explanted calvaria (wEx) and the defined sagittal suture (sS) and coronal suture (cS) (*top image*) revealed significant increase in samples with rNell-1 treatment (*bottom bar graph*) (**p < .01). (*J*) Three major MAPK pathways, ERK, JNK, and p38, were tested for activation status on rNell-1 stimulation of *Runx2^+/−^* or *Runx2^−/−^* NMCCs. rNell-1 stimulation intensified the phosphorylation of both ERK1/2 and JNK1 starting at 10 minutes in *Runx2^+/−^* and *Runx2^−/−^* NMCCs, but prolonged ERK1/2 and JNK1 phosphorylation was present only in *Runx2^+/−^* and not *Runx2^−/−^* NMCCs. (*K*) rNell-1 markedly increased the level of phosphorylated Runx2 (WB: p-Ser band at top) after 1 hour of stimulation with band-density analysis (*graph at bottom*). (*L*) Relative Runx2 activity in both *Runx2^+/−^* and *Runx2^−/−^* NMCCs was assessed using a 6OSE2 luciferase reporter construct. Relative luciferase activity was significantly higher in rNell-1- versus PBS-treated NMCCs when cotransfected with Runx2 expression plasmid (*dark blue and dark yellow in pRunx2/6OSE*). Higher luciferase activity also was induced by rNell-1 on *Runx2^+/−^* calvarial cells when cotransfected with pcDNA3.1 empty vector (*dark yellow in pcDNA3.1/6OSE*), whereas only background luciferase activity, similar to that from cells without 6OSE2 transfection (No treatment), was noticed on *Runx^−/−^* calvarial cells when cotransfected with empty vector (*dark blue in pcDNA3.1/6OSE*). *p < .05 versus corresponding PBS group.

Since a global Nell-1 expression model partially rescued the CCD phenotype in *Runx2*^*+/−*^ mice, to further confirm that the observed partial rescue of the CCD phenotype in vivo is attributable to Nell-1 activity in osteoblastic cell lineages, calvarial explants from *Runx2*^*+/−*^ and *Runx2*^*−/−*^ newborn mice were cultured in the presence (Fig. 4*A–C*, *G*) or absence (Fig. 4*D–F*, *H*) of recombinant (r)Nell-1 to create an environment without systemic influences. In general, *Runx2*^*+/−*^ explants stimulated with rNell-1 contained (Fig. 4*A–C*) more mineralization (as detected by calcein incorporation fluorescence) than explants cultured in the absence of rNell-1 (Fig. 4*D–F*). Specifically, rNell-1 stimulated significantly increased calcein uptake at actively extending osteogenic fronts of parietal bone plates (Fig. 4*A*). Histologic sections of sagittal and coronal sutures revealed markedly increased mineralization accompanied by impending sagittal suture fusion (Fig. 4*B*) and actual coronal suture fusion (Fig. 4*C*). Normally, wild-type mice sagittal and coronal sutures are expected to remain patent.(25) rNell-1 treatment also increased the thickness and size of parietal bones when compared with PBS vehicle control (Fig. 4*D–F*). Meanwhile, the control calvarias exhibited minimal mineralization within the sagittal suture mesenchyme (Fig. 4*E*) and between the overlapping frontal and parietal bone plates comprising the coronal suture (Fig. 4*F*). Consistent with the lack of Nell-1 rescue of bone formation in *Runx2*^*−/−*^ mice, *Runx2*^*−/−*^ calvarial explants cultured with or without rNell-1 did not exhibit any significant differences in alizarin red staining (Fig. 4*G, H*). Furthermore, the mineralization intensity increased by 46% overall with the presence of rNell-1 compared with the vehicle group when whole explanted calvaria were measured (wEx). There was a slightly higher increase, at 63% and 55%, in defined sagittal and coronal suture (sS and cS) regions of the samples with rhNell-1 treatment over vehicle control, respectively (Fig. 4*I*). Taken together, these ex vivo studies clearly demonstrated that Nell-1 was sufficient to promote mineralization and bone formation (eg, suture fusion) in *Runx2*^*+/−*^ mice containing committed preosteoblast cells but insufficient to promote mineralization in *Runx2*^*−/−*^ mice containing undifferentiated mesenchymal cells. These data indicate that Nell-1 is not sufficient to promote bone formation in the absence of Runx2 and that some level of Runx2-dependent factors or Runx2 itself is required for Nell-1 to promote full osteoblastic differentiation.

To investigate the underlying mechanisms of *Nell-1* responsiveness in *Runx2*^*+/−*^ backgrounds and lack thereof in *Runx2*^*−/−*^ backgrounds, the activation of three major MAPK pathways, including ERK, JNK, and p38, and Runx2 phosphorylation status were investigated using *Runx2*^*+/−*^ and/or *Runx2*^*−/−*^ NMCCs. Currently, it is known that phosphorylation can modulate Runx2 activity, and increased Runx2 phosphorylation has been observed after rNell-1 stimulation of rat fetal calvarial cells.(26,27) Our data indicated that rNell-1 stimulation markedly intensified phosphorylation of both ERK1/2 and JNK1 starting at 10 minutes in both *Runx2*^*+/−*^ and *Runx2*^*−/−*^ cells, but phosphorylation was higher and more sustained in *Runx2*^*+/−*^ cells than in *Runx2*^*−/−*^ cells (Fig. 4*J*). Interestingly, phosphorylated p38 (p-p38) levels were consistently high and did not change much on Nell-1 stimulation in either *Runx2*^*+/−*^ or *Runx2*^*−/−*^ cells (Fig. 4*J*). The *Runx2*^*+/+*^ NMCCs exhibited similar ERK and JNK pathway phosphorylation patterns as that of *Runx2*^*+/−*^ NMCCs in response to rNell-1 stimulation (Supplemental Fig. S1*C* and Fig. 4*J*). Meanwhile, rNell-1 increased phosphorylated Runx2 levels (WB: p-Ser) in *Runx2*^*+/−*^ NMCCs (Fig. 4*K*). Furthermore, Nell-1 significantly increased the transactivating activity of Runx2 (Fig. 4*L*). Relative luciferase activity using the 6OSE2 reporter plasmid (6OSE) was significantly higher in *Runx2*^*+/−*^ NMCCs treated with rNell-1 (versus PBS) when cotransfected with empty vector (pcDNA3.1) and in both *Runx2*^*+/−*^ and *Runx2*^*−/−*^ NMCCs when treated with rNell-1 (versus PBS) and cotransfected with Runx2 expression plasmid (pRunx2). Overall, a dose-dependent effect on 6OSE activity was observed with increasing rNell-1 application (Supplemental Fig. S1*D*). These data indicate that *Nell-1* responsiveness in *Runx2*^*+/−*^ but not *Runx2*^*−/−*^ backgrounds may result from sustainable and strong activation of ERK and JNK pathways and enhanced phosphorylation of, in part, serine residues on Runx2 that results in increased Runx2 activity.

### *Nell-1* knockdown partially blocked Runx2-induced osteoblastic differentiation of NMCCs

**Fig. 5:**
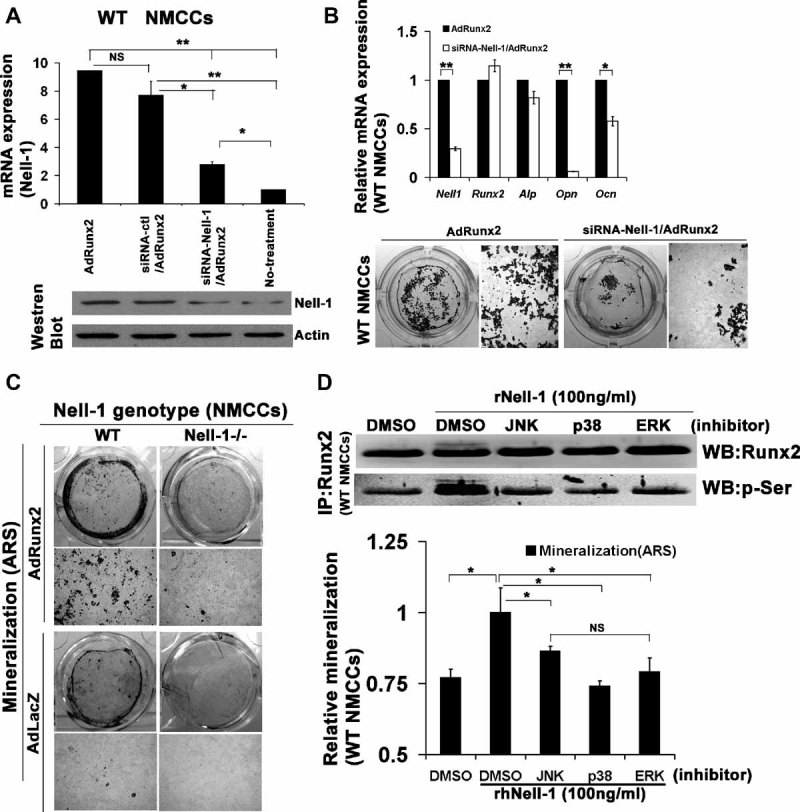
Nell-1 is critical for Runx2-induced osteoblastic differentiation in NMCCs. (*A*) Real-time RT-PCR *Nell-1* expression (*top bar graph*) 48 hours after siRNA^Nell-1^ and unrelated negative control siRNA transfection and/or 24 hours after Ad*Runx2* transduction. Ad*Runx2* significantly increased *Nell-1* transcripts in calvarial cells up to 9.5-fold, whereas siRNA^Nell-1^ transfection decreased the Ad*Runx2*-mediated *Nell-1* response by approximately 3-fold. No-treatment control revealed low basal *Nell-1* expression. In agreement with the real-time RT-PCR *Nell-1* expression data, *siRNA^Nell-1^* transfection also blocked upregulation of Nell-1 protein by Ad*Runx2* 72 hours after transduction (*bottom image of Western blot*). (*B*) Real-time RT-PCR (*top bar graph*) of relative *Nell-1*, *Runx2*, *Opn*, *Ocn*, and *Alp* levels 48 hours after siRNA transfection and/or 24 hours after Ad*Runx2* transduction. *siRNA^Nell-1^* transfection significantly abrogated Ad*Runx2-*associated upregulation of *Nell-1*, *Opn*, and *Ocn* but not *Alp* (*p < .05; **p < .01). Von Kossa staining (*bottom images*) of NMCCs on day 18 showed that *siRNA^Nell-1^* transfection significantly inhibited mineralization of Ad*Runx2-*transduced calvarial cells (*whole-plate image at left and higher-magnification images at right of each panel*). (*C*) Ad*Runx2* transduction promoted mineralization (ARS = alizarin red staining) in wild-type calvarial cells but not in *Nell-1^−/−^* calvarial cells from ENU-induced *Nell-1*-deficient mice (*whole plate image at top and higher-magnification image at bottom of each panel*). (*D*) rNell-1 significantly enhanced Runx2 phosphorylation (WB: p-Ser) (*top image, first two lanes*). All three MAPK inhibitors partially blocked Nell-1-associated Runx2 phosphorylation and mineralization (ARS) in *Runx2^+/+^* NMCCs (*lower bar graph*). *p < .05; NS = no significance.

Having established that Nell-1 is sufficient to promote mineralization and bone formation (eg, suture fusion) in partially deficient Runx2 states (eg, *Runx2*^+/*−*^), the next step was to determine whether normal Runx2*-*induced osteoblastic differentiation (eg, *Runx2*^*+/+*^) requires Nell-1. Wild-type (*Runx2*^*+/+*^) NMCCs express endogenous *Nell-1* at relatively low levels, but *Nell-1* expression is rapidly induced on *Runx2* stimulation. To establish whether Nell-1 blockade will inhibit Runx2-mediated osteogenic differentiation, wild-type NMCCs were pretransfected with *Nell-1* siRNA (siRNA^Nell-1^) or unrelated negative control siRNA 24 hours prior to Ad*Runx2* infection and compared with Ad*Runx2* transduction alone and no treatment controls (Fig. 5*A, B*). Real-time RT-PCR and Western blot analyses confirmed that only siRNA^Nell-1^ significantly blocked *Runx2*-induced production of *Nell-1* transcripts and protein. Quantitatively, *Nell-1* mRNA was 9.5-fold higher in NMCCs transduced with Ad*Runx2* compared with control NMCCs. Up to 70% of the upregulated *Nell-1* mRNA was significantly knocked down using siRNA^Nell-1^ treatment (Fig. 5*A*). Functionally, siRNA^Nell-1^ treatment significantly decreased *Opn* and *Ocn* expression by 80% and 50%, respectively, without significant effects on *Alp* or *Runx2* transcription (*p* > 0.05; Fig. 5*B*, *upper panel*). Meanwhile, NMCCs transduced with Ad*Runx2* revealed more prominent and thicker mineralized clusters, as detected by von Kossa staining, whereas siRNA^Nell-1^ addition substantially inhibited mineralization in the *AdRunx2-*transduced NMCCs (Fig. 5*B*, *lower panel*). Consistent with the *Alp* mRNA data in Fig. 5*B*, Alp activity of NMCCs on days 3, 6, and 9 after transfection with *Nell-1* siRNA with or without Ad*Runx2* revealed only minor alterations on days 6 and 9 (data not shown).

Notably, the transduction of Ad*Runx2* enhanced mineralization [as assessed by alizarin red staining (ARS)] on wild-type (ie, *Nell-1*^*+/+*^) calvarial cells but not on *Nell-1*^*−/−*^ calvarial cells derived from ENU-induced *Nell-1-*deficient mouse (Fig. 5*C*), indicating that Nell-1 is required for Runx2's function on terminal osteoblast differentiation and mineralization. In addition, rNell-1 significantly increased phosphorylated Runx2 (WB: p-Ser) in *Runx2*^*+/+*^and *Runx2*^*+/−*^ NMCCs. Meanwhile, all three MAPK inhibitors (JNK, p38, and ERK) partially blocked Runx2 phosphorylation and decreased Nell-1-induced mineralization (Fig. 5*D*). However, the inhibitors of JNK (SP600125) and ERK (PD90589) also decreased cell proliferation (Supplemental Fig. S1*E*) and increased apoptosis when added alone (Supplemental Fig. S2) in NMCCs. Thus nonspecific toxic effects from the inhibitors also may have contributed to the observed suppression of Nell-1-induced mineralization.

Collectively, these data show that *Nell-1* knockdown by siRNA^Nell-1^ or intrinsic *Nell-1* deficiency (*Nell-1*^*−/−*^ from ENU-induced mutation) functionally compromises *Runx2*-induced *Opn* and *Ocn* expression and ECM mineralization—confirming that Nell-1 is a significant functional mediator of downstream Runx2 activity. In addition, the enhanced Runx2 phosphorylation through activation of MAPK pathways by Nell-1 is one of the mechanisms underlying the functional relationship between Runx2 and Nell-1.

### Nell-1 partially restored osteoblastic differentiation in *Runx2* null NMCCs

**Fig. 6:**
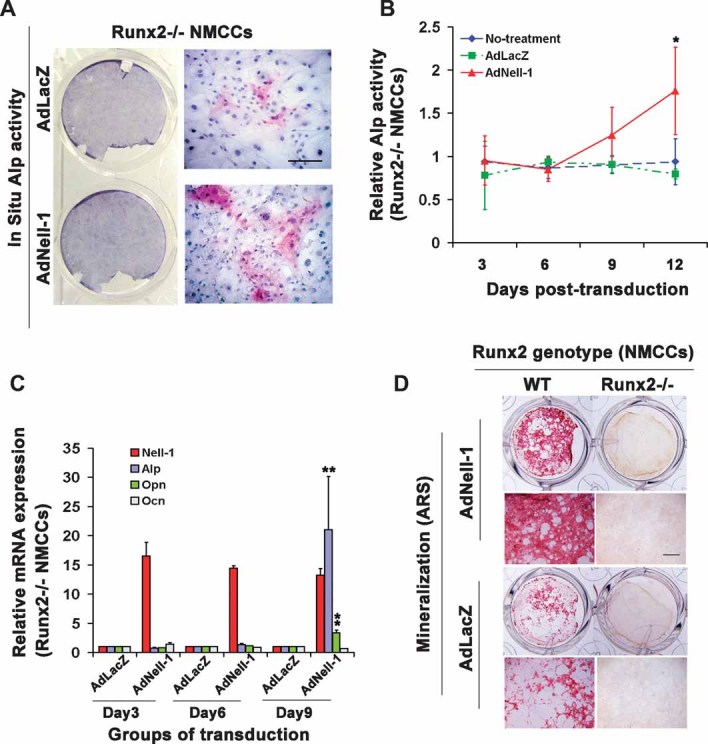
Nell-1 affects *Runx2^−/−^* NMCC differentiation. (*A*) Alp histochemistry on *Runx2^−/−^* NMCCs transduced with Ad*Nell-1* or Ad*LacZ.* Note increased Alp staining on day 9 after Ad*Nell-1* transduction (*whole-plate image at left and higher-magnification images at right of each panel*). Scale bar: 50 µm. (*B*) Relative quantitation of Alp activity in *Runx2^−/−^* NMCCs transduced with Ad*Nell-1* (*red*) and Ad*LacZ* (*green*) or no-treatment (*blue*) control on days 3, 6, 9, and 12. *p < .05. (*C*) Graph displaying real-time RT-PCR results for *Nell-1*, *Alp*, *Opn*, and *Ocn* expression in *Runx2^−/−^* NMCCs transduced with Ad*LacZ* or with Ad*Nell-1* on days 3, 6, and 9. **p < .01. (*D*) Mineralization (ARS) of *Runx2^−/−^* and wild-type NMCCs transduced with Ad*Nell-1* or Ad*LacZ* on day 27 (*whole-plate image at top and higher-magnification image at bottom of each panel*).

Although *Nell-1* overexpression did not restore bone formation in the *Runx2*^*−/−*^*/CMV-Nell-1* mice and rNell-1 did not mineralize *Runx2*^*−/−*^ calvarial explants, it is of interest to determine whether Nell-1 can induce osteogenic differentiation in the absence of Runx2—because Nell-1 expression was not completely absent in *Runx2-*deficient animals (Fig. 2*A*, *F–H*). Osteoblastic marker gene expression, Alp activity, and mineralization were examined in *Runx2*^*−/−*^ NMCCs transduced with Ad*Nell-1* and Ad*LacZ*. Surprisingly, Alp activity was significantly increased in Ad*Nell-1-*transduced *Runx2*^*−/−*^ NMCCs on day 12 (Fig. 6*A, B*), and *Alp* transcripts were increased over 20-fold on day 9 (Fig. 6*C*). Notably, *Opn*, a slightly later marker of osteoblastic differentiation, also was increased fourfold on day 9, whereas *Ocn* remained unchanged (Fig. 6*C*). These data are distinctly different from those in *Runx2*^*+/−*^ animals, where *Opn*, *Ocn*, but not *Alp*, were significantly upregulated with in vivo Nell-1 compensation (Fig. 3*F–H*). Overexpression of *Nell-1*, however, was insufficient to induce mineralization in *Runx2*^*−/−*^ NMCCs even after 27 days of culture, whereas *Nell-1-*transduced *Runx2*^*+/+*^ NMCCs were highly mineralized (Fig. 6*D*). Taken together, these results show that *Nell-1* overexpression in *Runx2*^*−/−*^ NMCCs can induce a certain level of osteogenic differentiation independent of Runx2 (as evidenced by initial *Alp* and then *Opn* expression), but Nell-1 alone is insufficient to promote terminal osteoblastic differentiation (ie, mineralization) in *Runx2*^*−/−*^ NMCCs cells. This accounts for the inability of Nell-1 to rescue mineralized bone formation in *Runx2*^*−/−*^ mice.

## Discussion

Commitment of undifferentiated mesenchymal stem cells to an osteoprogenitor lineage is first marked by expression of Runx2.(1,2) Furthermore, most described osteoinductive factors appear to converge mechanistically at Runx2 as well. Examples include bone morphogenetic proteins (BMP) 2 and 7, insulin-like growth factor (IGF-1), and transforming growth factor (TGF) β1, which are known to functionally modulate Runx2.(1,28–30) Meanwhile, fibroblast growth factor (FGF) 2 may increase Runx2 activity through a mitogen-activated protein kinase (MAPK) pathway, whereas parathyroid hormone (PTH) may increase Runx2 activity through protein kinase A and C (PKA and PKC) pathways.(27,31) In addition, Javed and colleagues have demonstrated recently that RUNX2 is a critical molecular end point for execution and completion of TGF-β/BMP signaling in osteoblasts.(32) Overall, Runx2 is essential for osteoblast formation and function because it is expressed by all osteoblasts irrespective of embryonic origin or mode of ossification.(3) However, the exact mechanism by which Runx2 exerts its activity on osteoblasts and chondrocytes is largely unknown. Except for osterix (Osx), another transcription factor that is absolutely required for osteoblast formation,(11,33) attempts thus far to identify critical downstream functional mediators of Runx2 in mineralization and bone formation have not been successful. We previously established that the human *NELL-1* gene is directly regulated by Runx2(15) and that *Nell-1-*deficient mice exhibit severe axial and appendicular skeletal anomalies(16)—indicating that Runx2-regulated Nell-1 is required for normal skeletogenesis. We show here that Nell-1 is a critical downstream Runx2 functional mediator.

In this study, we verified on multiple levels that mechanistically, Runx2 exerts many of its effects on osteoblasts through Nell-1. Specifically, (1) temporally and spatially, *Nell-1* expression correlated closely with endogenous *Runx2* expression in calvarial and long bone tissues in wild-type and *Runx2-*deficient mice, (2) cross-mating *CMV-Nell-1* mice with *Runx2*^*+/−*^ mice partially rescued the CCD-like calvarial defects phenotype, (3) rNell-1 protein added to mineralization-defective *Runx2*^*+/−*^ calvarial explants induced mineralization and bone formation at sagittal and coronal sutures, (4) rNell-1 protein increases ERK1/2 and JNK1 phosphorylation, which is followed by increased Runx2 phosphorylation and activity in a dose-dependent manner, (5) Runx2-mediated osteoblastic differentiation and mineralization was significantly reduced by transfection of *Nell-1* siRNA to *Runx2*^*+/+*^ NMCCs and in ENU-mutated *Nell-1*^*−/−*^ NMCCs, and (6) Ad*Nell-1* partially rescued osteoblastic gene expression but not mineralization in newborn calvarial cells from *Runx2*^*−/−*^ mice. Collectively, these data demonstrate that Nell-1 is a critical downstream Runx2 functional mediator insofar as Runx2-regulated Nell-1 promotes osteoblastic differentiation through, in part, activation of MAPK and enhanced phosphorylation of Runx2 and that Runx2 activity is significantly reduced when *Nell-1* is blocked or absent.

In our previous report, NELL-1 localized to more mature osteoblasts at the osteogenic front in fusing and newly fused sutures from UCS patients.(14) This study confirmed a close correlation between levels of endogenous Runx2 and Nell-1 expression during development at the tissue level (eg, lower Nell-1 in long bone versus calvaria) and genotype levels (eg, higher Nell-1 in *Runx2*^+/+^ versus *Runx2*^+/*−*^), which suggests that the multifunctional OSE2 sites on the *Nell-1* promoter described previously(15) are highly relevant to controlling *Nell-1* transcription in vivo. The CCD-like calvarial defect phenotype of *Nell-1-*deficient mice further indicates that Nell-1 has a critical role in the Runx2 osteoblastic differentiation pathway.

The importance of Nell-1 as a downstream mediator of Runx2 activity was clearly demonstrated when siRNA inhibition of *Nell-1* significantly reduced Runx2-induced mineralization of *Runx2*^*+/+*^ NMCCs as well as Runx2-induced upregulation of *Opn* and *Ocn* but not necessarily *Alp*. In addition, a significant reduction in Runx2-induced mineralization also was observed in *Nell-1-*deficient NMCCs. These data are consistent with Nell-1 being an important downstream Runx2 mediator that preferentially promotes late rather than early osteoblastic differentiation in the presence of Runx2. The inability of Nell-1 to induce bone formation in *Runx2*^*−/−*^ NMCCs is of interest. In particular, BMP-2 treatment of *Runx2* null cells also failed to induce complete osteogenic differentiation. Komori and colleagues were able to induce increased Alp activity and low-level Ocn expression in *Runx2*^*−/−*^ calvarial cells at pharmacologic dosages of rhBMP-2 above 300 ng/mL; however, they noted that those cells did not form bone and were not considered mature osteoblasts.(34) Similarly, long-term culture of E17.5 *Runx2*^*−/−*^ mouse calvarial cells in osteogenic medium or with BMP-2 also failed to induce terminal osteoblastic differentiation.(35) Meanwhile, we demonstrated that *Runx2*^*−/−*^ NMCCs infected with adenoviral Nell-1 displayed increased Alp transcripts and activity and, to a lesser degree, *Opn* transcripts during osteoblastic differentiation in vitro. Increased Alp or Opn expression demonstrates that *Runx2*^*−*/*−*^ NMCCs are responsive to Nell-1 stimulation and that Nell-1 can induce a certain degree of osteoblastic differentiation in these cells through Runx2-independent pathways; Runx2, however, is still absolutely required for complete osteoblastic differentiation.

Mechanistically, our previous data indicated that Nell-1 induces osteoblastic differentiation in rat and human osteoblasts by activating MAPK pathways as well as promoting Runx2 phosphorylation.(26) In this study, we evaluated the responsiveness of MAPK pathways in NMCCs with different *Runx2* genotypes (+/ +, +/–, and –/–) and the changes in Runx2 phosphorylation/transactivating activity on rNell-1 stimulation. Surprisingly, *Runx2*^*−/−*^ NMCCs respond to rNell-1 stimulation as rapidly as *Runx2*^+/*−*^ and *Runx2*^*+/+*^ NMCCs do with respect to ERK and JNK pathway activation. However, the intensity and sustainability of ERK1/2 and JNK1 activation in *Runx2*^*−/−*^ NMCCs were severely reduced. In addition, Runx2 phosphorylation and transactivating activity were enhanced on rNell-1 stimulation in *Runx2*^*+/+*^ and *Runx2* ^+/*−*^ NMCCs. In contrast, *Runx2*^*−/−*^ NMCCs exhibited increased Runx2 phosphorylation and transactivating activity by rNell-1 stimulation only after introduction of exogenous Runx2 by transfection. Meanwhile, although rNell-1 stimulation activated ERK1/2 and JNK1 but not p38, the inhibition of ERK, JNK, or p38 separately reduced Runx2 phosphorylation and mineralization in rNell-1-treated *Runx2*^*+/+*^ NMCCs. These data suggest that Nell-1 promotes osteoblastic differentiation partially through MAPK pathways that cross-talk with Runx2 (eg, Nell-1 increases Runx2 phosphorylation and transactivating activity) and pathways that do not necessarily cross-talk with Runx2 (eg, Nell-1 increases osteoblastic markers Alp and *Opn* expression in *Runx2*^*−/−*^ NMCCs).

Runx2 phosphorylation is thought to be crucial for its activity.(27) We postulate a mutually dependent mechanism between Runx2 and Nell-1 based on the fact that Runx2 directly regulates Nell-1 transcription(15) and Nell-1 significantly modulates Runx2 activity during osteoblastic differentiation. Therefore, Nell-1 can induce osteoblastic differentiation by at least one of two mechanisms: (1) enhancing overall Runx2 phosphorylation and activity (Runx2-dependent) and (2) other effects not related to modulating Runx2 activity (Runx2-independent). Conspicuously, ENU-induced *Nell-1-*deficient mice, while exhibiting similar CCD-like calvarial phenotypes as *Runx2*^*+/−*^ mice, also display rib cage vertebral abnormalities not described in *Runx2*^*+/−*^ mice.(16) This, coupled with our current data demonstrating Nell-1 induction of Alp and *Opn* expression in *Runx2*^*−/−*^ NMCCs, indicates that a subset of Nell-1 effects is not necessarily related to Runx2 modulation.

Although Runx2 may exert some of its effects mechanistically through Nell-1 and vice versa, the exact mechanism by which Nell-1 can promote osteoblast differentiation through effects not related to modulating Runx2 activity is unclear. Structurally, NELL-1's N-terminal domain contains a laminin G–like domain [previously known as an N-terminal thrombospondin 1 (TSP-1)–like module] that likely interacts with heparan sulfate proteoglycans and integrin-related molecules.(17,26) The EGF-like domains of rat Nell-1 are phosphorylated by protein kinase C (PKC) β1,(36) but it is unknown at this point whether Nell-1 phosphorylation increases or decreases its activity and whether binding to PKC changes Nell-1's intracellular distribution or function or its secretion into the extracellular matrix. In addition, MAPK may not be the only pathway by which Nell-1 promotes Runx2 phosphorylation. Given the fact that Nell-1 contains TSP-N and EGF-like repeat domains, other pathways for promoting Runx2 phosphorylation may involve Nell-1–integrin interactions involving focal adhesion kinase (FAK) and PKC or calcium-binding–mediated kinase activation.(26)

*NELL-1* was originally found as a local factor with upregulation at fusing and fused sutures from UCS patients. Subsequently, we discovered that *NELL-1* transcription is tightly regulated by Runx2, a key mechanistic convergence point for CS development. Interestingly, while syndromic and nonsyndromic CS differ in extracraniofacial presentation and in the pattern and degree of suture involvement, the histomorphometric phenotype at the level of the pathologic closed/closing suture is virtually indistinguishable.(37) This implies that even widely disparate regulatory factors such as fibroblast growth factor receptors (FGFRs) 1, 2, and 4 and Twist causing distinctly different CS syndromes nonetheless may converge mechanistically at the level of the calvaria to affect suture fusion. In fact, altered FGFRs and Twist activity largely associate with downstream modulation of Runx2 expression and/or activity—making Runx2 a potentially key molecule of mechanistic convergence for CS.(38,39) Overall, Runx2's activities are etiopathologically involved in many congenital craniofacial anomalies, including CCD, in humans caused by Runx2 mutations.(4,5)

The identification of Nell-1, a secretory molecule, as a key component of the Runx2-mediated bone-formation network opens up exciting possibilities for future NELL-1-blocking therapies to treat CS or other conditions involving undesirable bone formation (eg, heterotopic ossification). On the other hand, use of NELL-1 as an osteoinductive molecule may have even wider applications than anti-NELL-1 therapeutics. Thus far we have shown comparable Nell-1- versus BMP-induced bone regeneration in multiple animal models from rat palatal distraction,(18) calvarial defect,(23) and spinal fusion(40) to sheep spinal fusion.(41) More notably, Nell-1, by virtue of being transcriptionally “downstream” of Runx2, is a more highly selective osteoinductive molecule in vivo than BMP-2(20) and is capable of inducing high-quality bone regeneration from BMSCs.(19) The development of more osteoinductive growth factors with divergent but complementary bone-formation pathways can serve to maximize biologic efficiency—which, in turn, may improve clinical efficacy, lower dose requirements (and costs), and minimize potential adverse effects of current osteoinductive therapeutics.
