# Supplementary figures and images for: Nell-1, a key Functional Mediator of Runx2, Partially Rescues Calvarial Defects in Runx2+/− Mice
Source: J Bone Miner Res. 2010 Oct 11;26(4):777–91. doi: 10.1002/jbmr.267 (PMC3179324; doi:10.1002/jbmr.267)

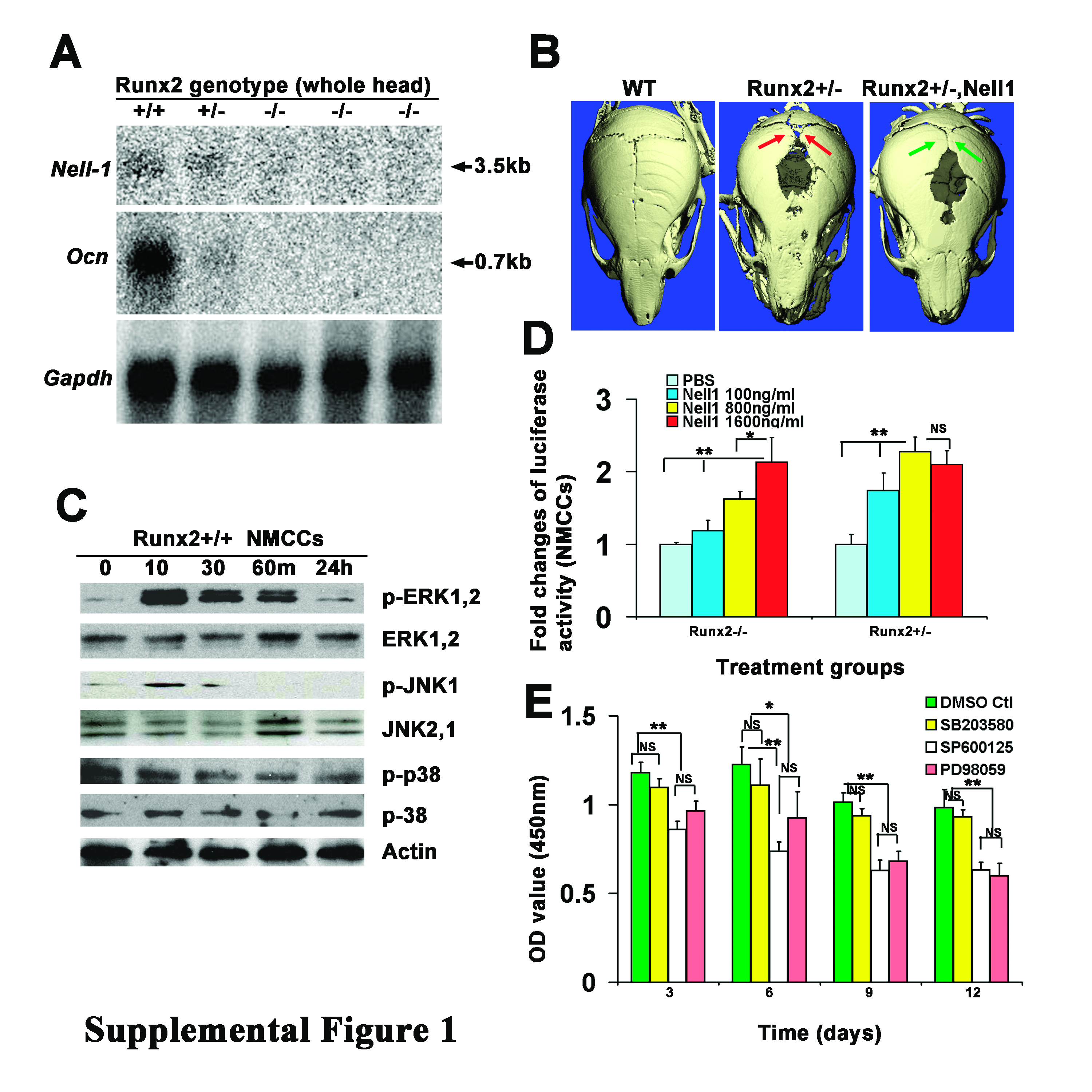

Supplement: Supplementary file 1 [file jbmr0026-0777-SD1.tif]
